# A bibliometric analysis of global research trends in adoptive cell therapy for triple negative breast cancer over the past 15 years

**DOI:** 10.1007/s12672-026-05277-6

**Published:** 2026-06-10

**Authors:** Wesam Ibrahim Abo Elenien, Nada Ashraf Ibrahim, Mariam Mohamed, Youssef Abo Elenien, Momen Heiba, Osama Abouelenin, Dunya Abbas Mahmood, Farhan Khaleel Hussein

**Affiliations:** 1 Faculty of Pharmacy, Alexandria University, Alexandria, Egypt; 2 Faculty of Science, Ain Shams University, Cairo, Egypt; 3 Faculty of Medicine, Alexandria University, Alexandria, Egypt; 4 Department of Chemistry, College of Education for Pure Sciences, University of Kirkuk, Kirkuk, Iraq; 5 Department of Biology, College of Education for Pure Sciences, University of Kirkuk, Kirkuk, 36001 Iraq

**Keywords:** Adoptive cell therapy, Bibliometric analysis, CAR-T cells, Immunotherapy, Tumor microenvironment, Triple-negative breast cancer, Tumor-infiltrating lymphocytes

## Abstract

**Background:**

The aggressive and diverse subtype of breast cancer known as triple-negative breast cancer (TNBC) has poor clinical outcomes and few specific therapeutic choices. Tumor-infiltrating lymphocytes (TILs), T-cell receptor-engineered T cells, and chimeric antigen receptor T (CAR-T) cells are examples of adoptive cell therapy (ACT), which has become a promising immunotherapeutic approach. Its clinical application in TNBC is still difficult, nevertheless. This study used bibliometric techniques to thoroughly assess growing hotspots, intellectual structure, and worldwide research trends pertaining to ACT in TNBC.

**Methods:**

The Scopus database was searched for publications related to ACT in TNBC from 2011 to 2025. There were only original articles and reviews written in English. VOSviewer (version 1.6.20) and Microsoft Excel 2021 were used to analyse bibliometric indicators, such as annual publication output, country and institutional contributions, authorship patterns, citation characteristics, and keyword co-occurrence. To investigate thematic evolution and collaboration patterns, network visualisation and clustering analysis were carried out.

**Results:**

With a compound annual growth rate of more than 60%, a total of 8,496 publications were found, indicating an exponential rise in research output, especially beyond 2020. Together, China and the US accounted for over 60% of all publications, dominating the world’s research output. The core research network was made up of a few institutions and very productive writers. CAR-T cell therapy, tumor microenvironment manipulation, immunological checkpoint inhibition, metabolic reprogramming, and biomarker-driven methods were among the clinically orientated themes that emerged from foundational and preclinical investigations, according to keyword analysis. The literature shows ongoing translational difficulties with regard to tumor heterogeneity, antigen instability, immunosuppressive microenvironments, and safety concerns in solid tumors, despite increased research activity.

**Conclusion:**

Over the past ten years, research on ACT in TNBC has grown significantly, reflecting both unmet clinical need and growing scientific interest. However, continuous efforts to overcome biological and translational constraints are highlighted by the concentration of scientific leadership and the conceptual move towards combination methods and next-generation engineering approaches. This bibliometric analysis offers a thorough picture of the state of the field and could direct future research, teamwork, and the creation of more potent ACT tactics for TNBC.

## Introduction

Breast cancer (BC) is among the most prevalent cancers in women globally, and it’s the second leading cause of cancer-related mortality. BC is anticipated to continue rising in future years in both incidence and mortality [1]. Environmental, hormonal, genetic, lifestyle, and nutritional factors all have an impact on breast cancer. The expression patterns of its three primary receptors, estrogen receptor (ER), progesterone receptor (PR), and human epidermal growth factor receptor 2 (HER2), define its molecular subtypes [2]. Triple-negative breast cancer (TNBC) is a subtype defined by the absence or reduced expression of the ER, PR, and HER2 receptors [3]. TNBC accounts for about 20% of newly diagnosed breast cancer cases. TNBC is very challenging due to its aggressive nature, heterogeneity, and lack of targeted therapies like hormone or HER2 treatments in the other subtypes [4]. TNBC is frequently associated with germline BRCA1 mutations. It’s highly resistant to chemotherapy, resulting in worse outcomes, higher relapse rates, and increased distant metastasis. Most cases of metastatic TNBC are relapses from earlier occurrences [5]. TNBC is highly invasive and has a tendency for visceral spread, most commonly to the brain, lungs, and liver [6]. There is currently no standardized TNBC treatment regimen; However, recent advances in molecular profiling, emerging targeted treatments such as PARP inhibitors, and immunotherapies, including adoptive cell therapy (ACT) approaches like CAR-T cells, TIL therapy, and engineered T cells, are reshaping TNBC management [7, 8].

This bibliometric analysis synthesises publication trends, authorship patterns, institutional contributions, citation networks, and topic evolution across time in order to thoroughly assess worldwide research activity linked to adoptive immunotherapy in triple-negative breast cancer (TNBC). The report offers a comprehensive overview of research productivity, significant contributors, and new research hotspots by methodically mapping the global scientific output in this quickly developing topic. The analysis intends to help clinicians, researchers, and oncology specialists identify current knowledge gaps, comprehend the translational trajectory of adoptive immunotherapy in TNBC, and direct future research directions and therapeutic development through this thorough evaluation of the scientific landscape.

Bibliometrics is a subfield of informatics that deals with quantitative and qualitative literature analysis, using the literary system and bibliometric features as the research object. The contour distribution, relationship, and clustering of the research field may be statistically measured using this methods [9], and it has emerged as a popular methodology for evaluating the influence, quality, and legitimacy of academic work [10, 11]. Specifically, the evaluation might focus on the contributions and impacts of different authors, countries/regions, organizations, disciplines, and journals; it can also examine the status, paths, and limits of research activities [12, 13]. The most popular bibliometric visualization program for data processing and visualization is VOSviewer [14].

## Methods

### Data collection and search strategy

This bibliometric study was performed to analyze the global research trend of adoptive cell therapy (ACT) in triple-negative breast cancer (TNBC). Relevant publications were obtained only from the Scopus database, given its wide coverage and usability for bibliometric exploration. To prevent inconsistency from daily database updates, the maximum date published was set to 2025 (the first day of Jan. 1, 2026), and the records were restricted to those published after January 2010. The query used in the Scopus advanced search interface was as follows:

(“triple negative breast cancer” OR TNBC) AND (“adoptive cell therapy” OR “CAR-T cells” OR “TIL therapy”) AND PUBYEAR > 2009 AND PUBYEAR < 2026AND (LIMIT-TO (DOCTYPE, “ar”) OR LIMIT-TO (DOCTYPE, “re”))AND (LIMIT-TO (LANGUAGE, “English”)).

**Fig. 1:**
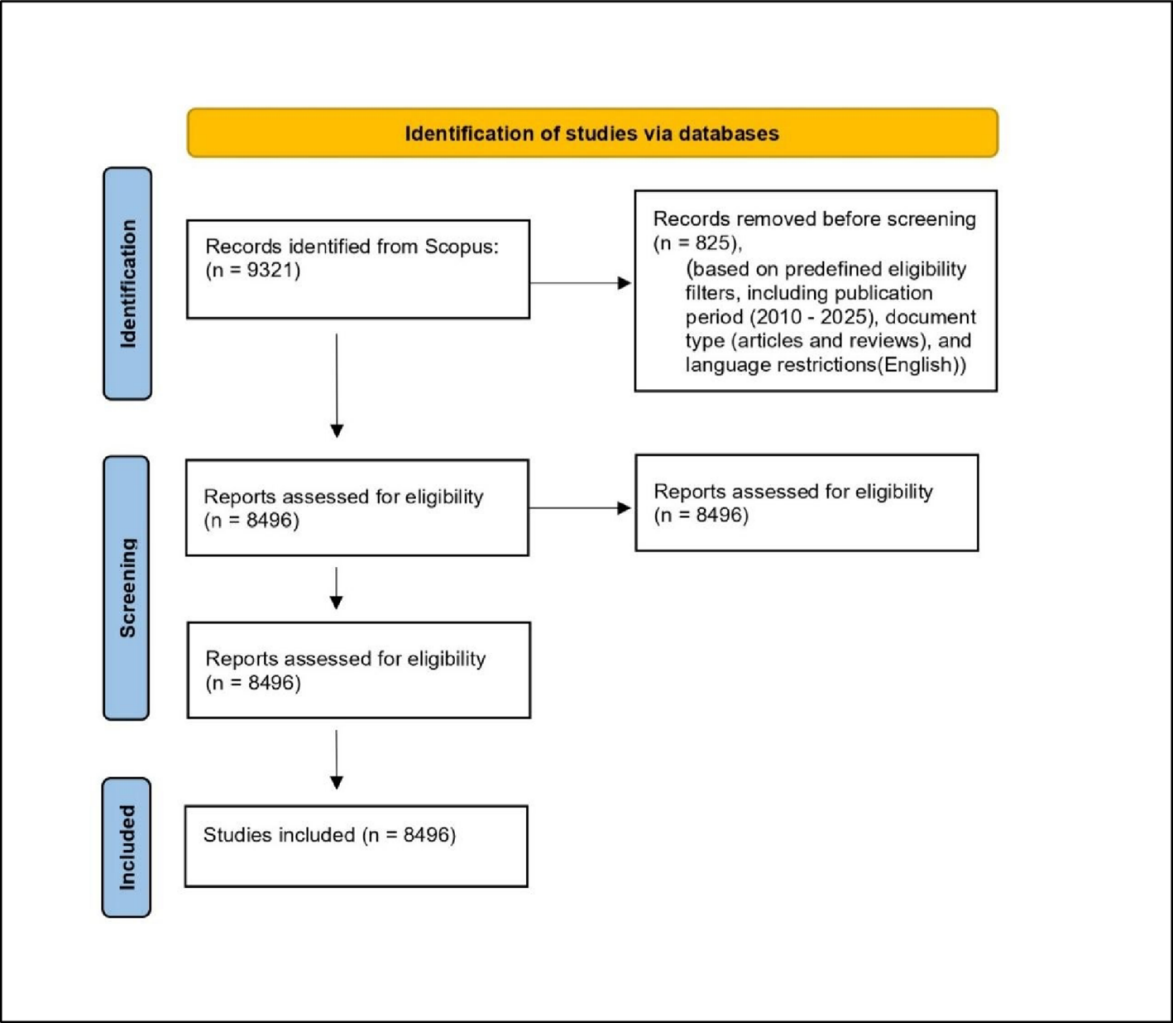
PRISMA flow diagram of study selection. No manual screening was performed, as all exclusions were applied automatically using database filters

Data on published articles between specified study periods, indexed in Scopus and written in English, both original and review articles, were included as inclusion criteria. Predefined database filters eliminated publications that were neither TNBC nor adoptive cell therapy-related, content not in English, and documents not of type: articles and reviews. The initial search retrieved 9321 records, and following application of the eligibility filters, 8496 publications remained. A PRISMA-style flowchart, as shown in Fig. 1, was used to illustrate the literature identification and selection process and to enhance transparency and reproducibility of the study.

### Data analysis and visualization

The bibliographic metadata of publications included in the documents were exported in CSV format and analyzed with VOSviewer (version 1.6.20) and Microsoft Excel 2021. Bibliometric mapping and visualization, including co-authorship, collaboration by country, and keyword co-occurrence analysis was performed using VOSviewer while Excel was used for descriptive analysis and organization of data. Moreover, the geographical distribution map organized in the manuscript is adapted from Insert Map feature of Microsoft Word based on publication data standardized at country level. The distribution of research productivity among countries was measured with the Herfindahl–Hirschman Index (HHI), calculated as HHI = Σ(s_i_²), where s_i_ is the share of publications produced by each country compared to the total number of included publications. Higher HHI values suggest a higher concentration of research output in the hands of fewer countries.

## Results

### Temporal distribution map of literature

**Fig. 2:**
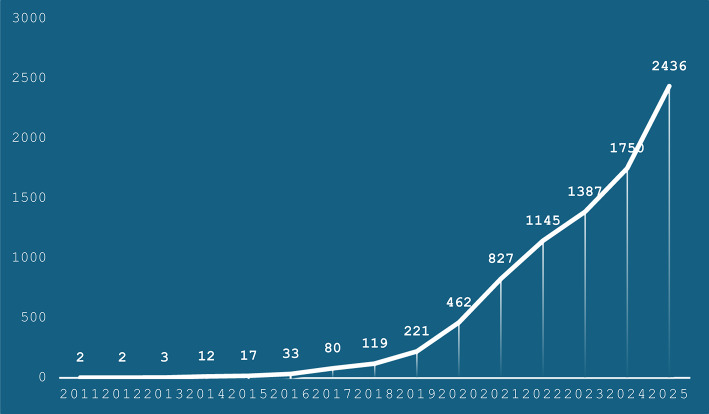
Annual publication output between 2011 and 2025

Over the course of the study period (2011–2025), there was a noticeable and consistent growth in research production, as indicated by the temporal distribution of publications. Figure 2 provides an illustration of this pattern. There was very little scholarly activity in the early years; in 2011 and 2012, there were only two publications per year. By 2016, there has been a gradual growth to 33 publications. The number of publications increased significantly starting in 2017, going from 80 in 2017 to 221 in 2019.

Then it was the so-called obvious ‘spike’, a post-2020 event. This suggests that research in this area was growing rapidly. The number of annual publications rose from 462 in 2020 to 827 in 2021. Growth would continue in the years ahead: 1,145 in 2022, 1,387 in 2023, and 1,750 in 2024. The number reached a high of 2,436 publications in 2025. In total, the annual number of publications increased by over 1,200 times from 2011 to 2025.

For the study period, the compounded annual growth rate (CAGR) was approximately 66.1%. This shows the extraordinarily fast development of the literature. The field maintained a positive growth trend even when the analysis was restricted to recent years (2019–2025). The CAGR of 49.2% implied a persistent and increasing level of interest in global research. The rapidly emerging status of the field 25, as compared to an early stage just a few years ago, is emphasized by its clear exponential growth.

### Distribution of countries

**Fig. 3:**
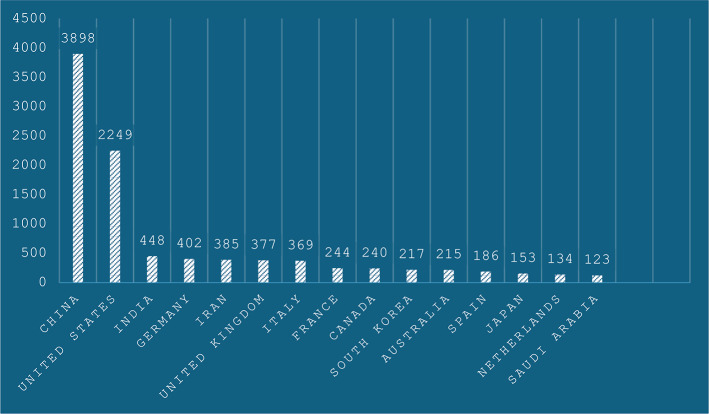
Top 15 countries with a higher number of publications

**Fig. 4:**
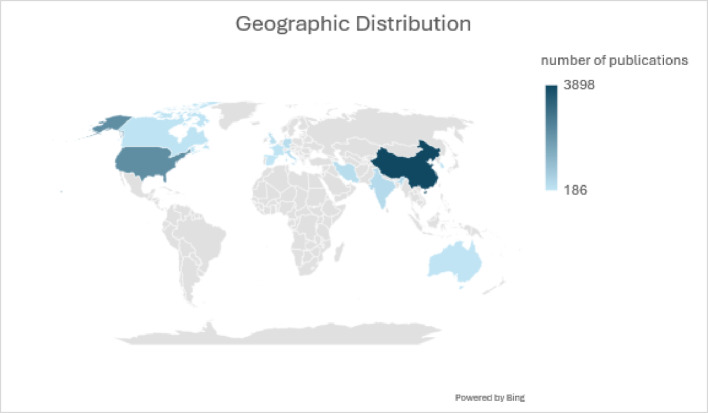
Geographic distribution of publications across Countries. The map was generated using the map chart feature in microsoft office (Microsoft Corporation, Redmond, WA, USA) based on country-level publication data extracted from Scopus; basemap data were provided through Bing Maps (Microsoft Corporation)

The regional distribution of articles shows that research output is highly uneven across countries. International publications predominantly came from 15 countries, as shown in Figs. 3 and 4, with China leading (*n* = 3898, ratio% = 38.4%). The U.S. was the second most productive country (2,249, 22.1%), reinforcing its considerable impact on the course of this research.

China and the US accounted for 60.5% of the publications, indicating a significant focus on research achievement. The Two-Country Concentration Ratio) CR₂ (for these two countries was greater than 60%, indicating that they had exclusive control. The top 5 countries contributing to research output were China, the US, India, Germany, and Iran (72.6% of the total output), with a sharp contrast between the top and bottom research producers across the globe.

The second most contributing country was India (*n* = 448, 4.4%), followed by Germany (*n* = 402, 4.0%), Iran (*n* = 385, 3.8%), the United Kingdom (*n* = 377, 3.8%), and Italy (*n* = 369, 3.7%). These were countries with consistent research output and from a variety of regions (Asia, Europe, and the Middle East).

France (*n* = 244, 2.4%), Canada (*n* = 240, 2.4%), South Korea (*n* = 217, 2.2%), and Australia (*n* = 215, 2.1%) also contributed significantly. Less research came from Spain (*n* = 186, 1.9%), Japan (*n* = 153, 1.5%), the Netherlands (*n* = 134, 1.3%), and Saudi Arabia (*n* = 123, 1.2%), although they were also contributing to the international literature.

The Herfindahl-Hirschman Index (HHI) for the country included publication output was 0.2, indicating a moderately high concentration of research activity. This is to say that most of the research leadership is still concentrated in a handful of countries, despite massive country involvement.

Taken together, the results reveal remarkable disparities. We report a large variation in research productivity across the globe that is likely to reflect differences in infrastructure, funding, and national priorities. The necessity of greater international collaboration and assistance to foster a more equitable global research environment is underscored by these findings.

**Fig. 5:**
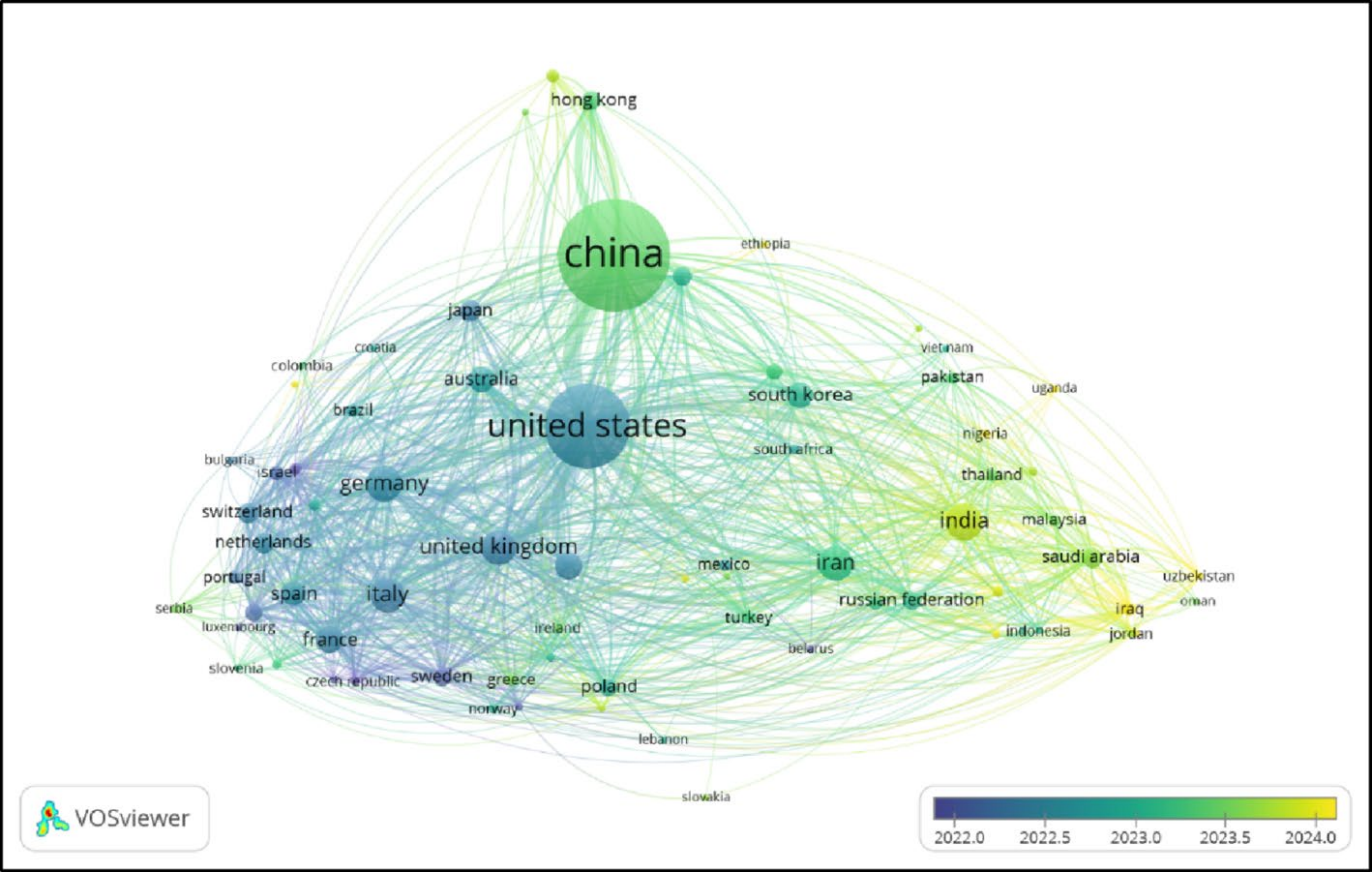
Overlay visualization map of Vosviewer

**Fig. 6:**
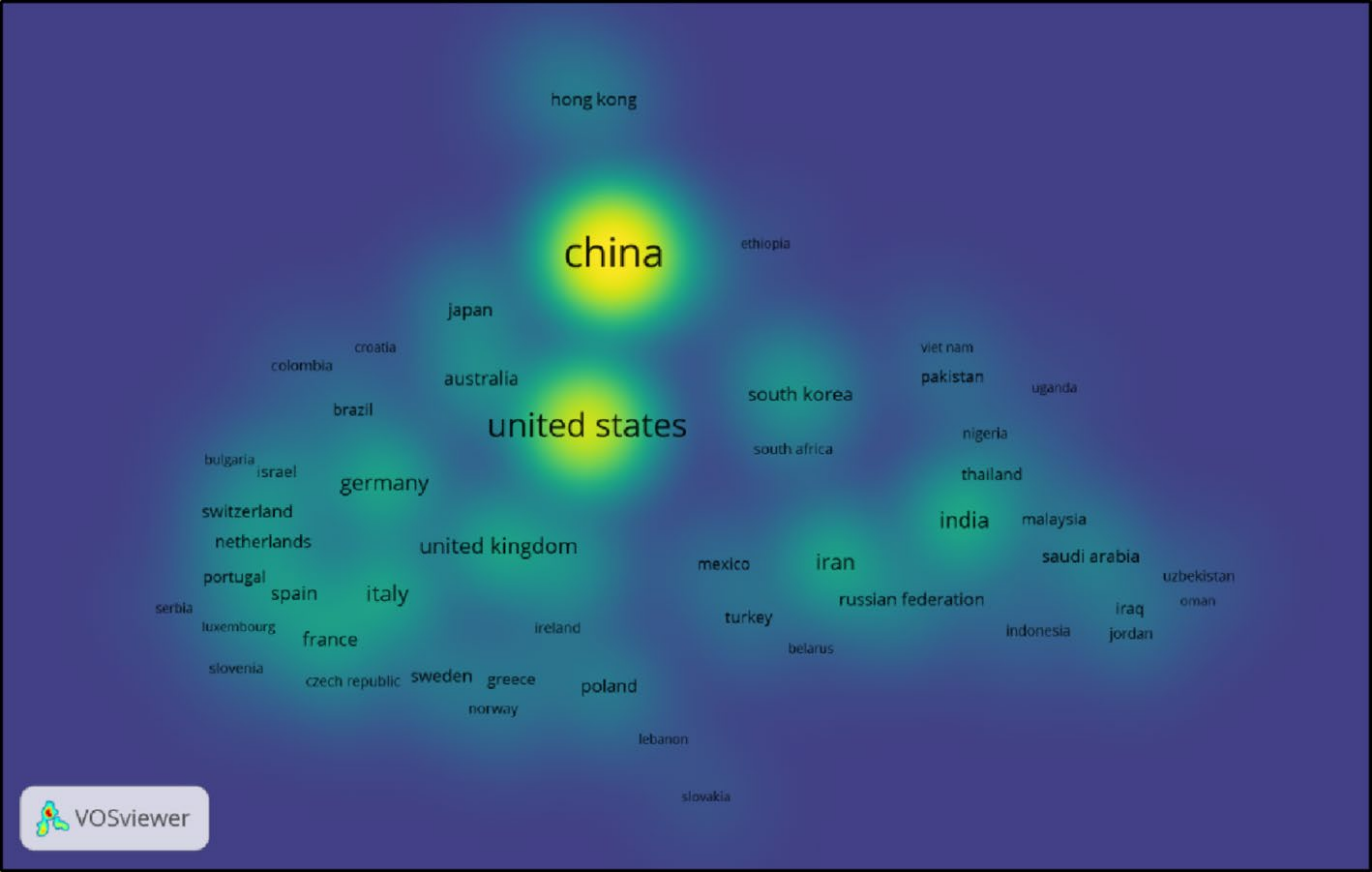
Density visualization map of Vosviewer

We also examined the distribution of publications by country and used VOSviewer to visualize international research cooperation. We configured the experiments to use modularity/LinLog and selected only countries with at least 5 documents. Of the 129 countries, 73 actually had that information and were featured in the visualization. VOSviewer clustered these countries into four colors. In the visualisations, larger nodes represent more publications, and thicker edges indicate stronger collaboration between countries. This clustering suggests that high-yield research countries, such as the United States of America, Japan, Italy, and China, are close (Fig. These collaboration networks and clusters are depicted in Figs. 5 and 6.

### Distribution of authors and research institutions

**Fig. 7:**
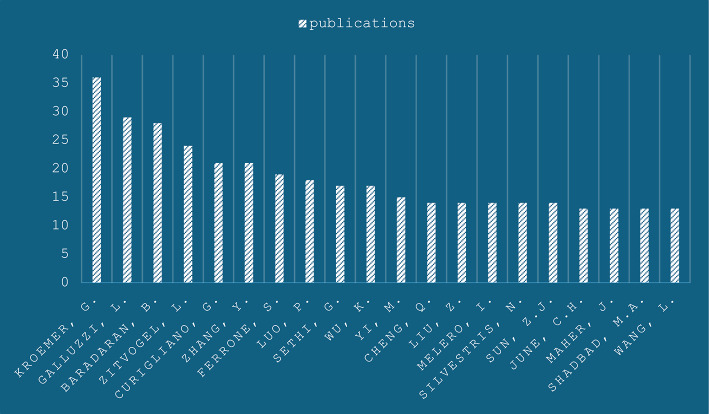
Top 20 authors with the highest number of publications

A significantly erroneous pattern can be seen in the distribution of author production, which is typical with established and quickly developing fields of study. Figure 7 illustrates that Kroemer, G. was the most prolific contributor with 36 publications, followed by Galluzzi, L. (*n* = 29) and Baradaran, B. (*n* = 28). These writers are important thought leaders who have made consistent and significant contributions to the area. Zitvogel, L. (*n* = 24), Curigliano, G. and Zhang, Y. (*n* = 21), and Ferrone, S. (*n* = 19) comprised a second tier of extremely productive authors. Additional notable contributors with persistent mid-to-high publishing activity were Luo, P. (*n* = 18), Sethi, G., and Wu, K. (*n* = 17).

Yi, M. (*n* = 15), Cheng, Q., Liu, Z., Melero, I., Silvestris, N., and Sun, Z.J. (each *n* = 14) made additional contributions, and June, C.H., Maher, J., Shadbad, M.A., and Wang, L. each provided 13 publications. A concentrated core of extremely productive scholars was indicated by the fact that the top 20 authors together accounted for a sizable part of all publications.

The observed distribution is consistent with Lotka’s law of scientific production, which asserts that although most academics publish fewer papers, a small percentage of writers contribute disproportionately to the literature. A core author group guiding research direction and subject extension is confirmed by the calculated author concentration ratio (CR² = 35.6%) for the top five authors, which demonstrates a high degree of knowledge production centralisation.

Overall, our results indicate that the field is marked by a strong set of nodes (very numerous) and edges (few prestigious authors), in which highly productive and sustained contributions are fairly rewarded. This organization is indicative of intellectual accretion around dominant research centres, as well as ongoing opportunities for the field to be advanced by new contributions.

**Fig. 8:**
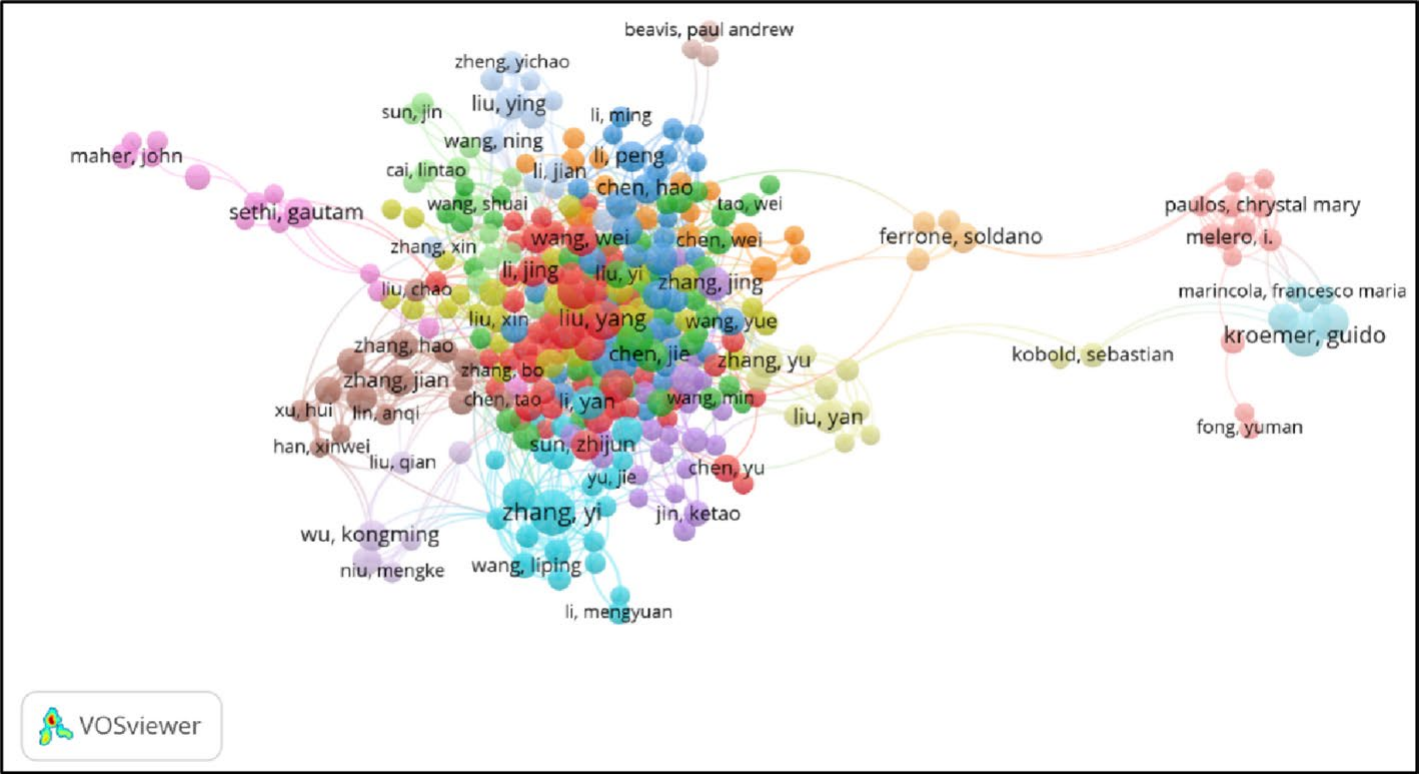
Network visualization map of authors’ distribution of Vosviewer

**Fig. 9:**
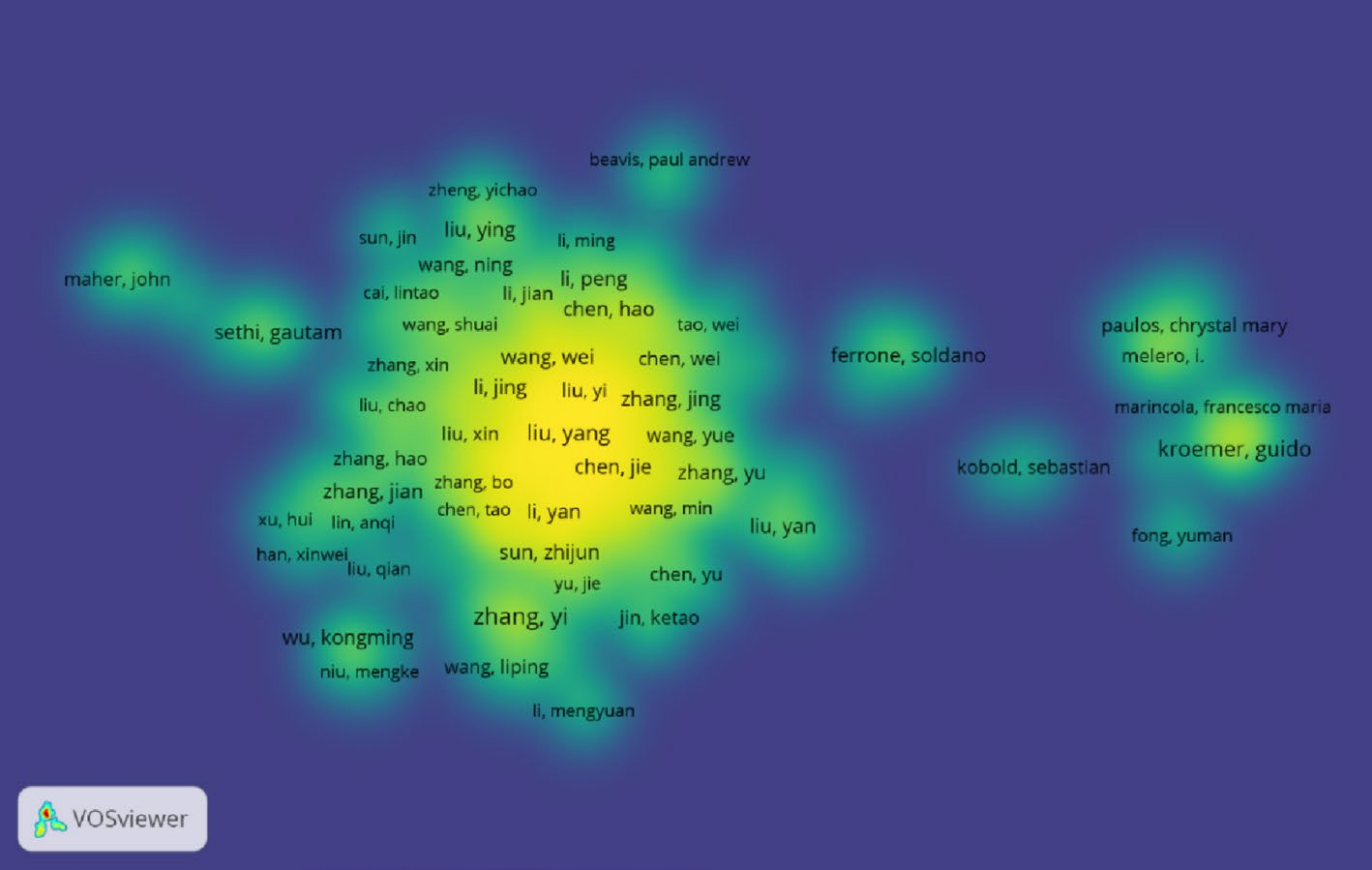
Density visualization map of authors’ distribution of VOSViewer

The co-authorship relations were extracted from VOSviewer for the investigation of collaboration. Modularity/LinLog was applied, and the minimum number of documents per author was set at 7. From 43,060 authors in the data set, we kept only 376 that met these requirements and were selected for visualization. These authors were grouped using the software VOSviewer, with a time overlay, in which the color of each node corresponds to the mean year of publication for that author. In the network and density map (Figs. 8 and 9), each node represents an author, and the linewidth indicates the number of papers they have published; nodes are connected by lines representing coauthorship relationships among scholars, with the weights indicating how many times two authors have co-published.

**Fig. 10:**
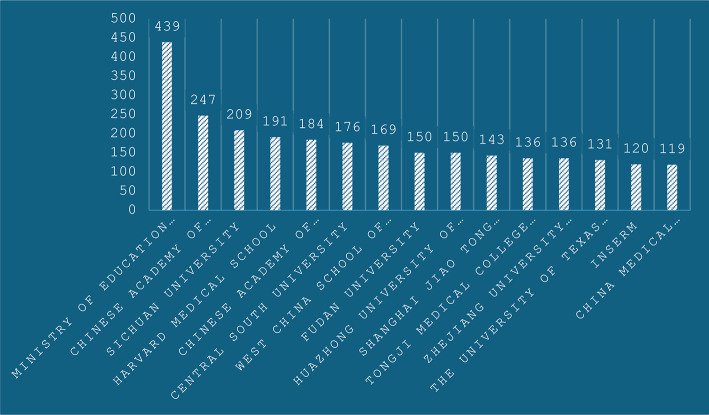
Top 15 institutions with the highest number of publications

The institutional distribution analysis revealed 15 top institutions that together produced 2,700 publications to the literature, as shown in Fig. 10. There was a strong institutional core in the discipline, as seen by the great concentration of research output among a few universities.

With 439 publications, or 16.26% of the total output, the Ministry of Education of the People’s Republic of China took first place. Third place went to Sichuan University with 209 publications (7.74%), followed by the Chinese Academy of Sciences with 247 publications (9.15%). Harvard Medical School was the most productive non-Chinese university, ranking fourth with 191 papers (7.07%). With 184 articles (6.81%), the Chinese Academy of Medical Sciences & Peking Union Medical College ranked second. A high institutional concentration ratio (CR²) was indicated by the top five institutions’ combined 1,270 publications, which accounted for 47.04% of all institutional production.

A second tier of productive institutions included Central South University (*n* = 176; 6.52%) and the West China School of Medicine/West China Hospital of Sichuan University (*n* = 169; 6.26%). Fudan University and Huazhong University of Science and Technology each contributed 150 publications (5.56% each), followed by Shanghai Jiao Tong University School of Medicine (*n* = 143; 5.30%).

Further contributions were observed from Tongji Medical College of Huazhong University of Science and Technology and Zhejiang University School of Medicine (each *n* = 136; 5.04%), The University of Texas MD Anderson Cancer Center (*n* = 131; 4.85%), Inserm (*n* = 120; 4.44%), and China Medical University, Shenyang (*n* = 119; 4.41%).

Eleven of the top 15 donors were from Chinese institutions overall, indicating a significant national concentration of research capacity. Although the field benefits from widespread institutional participation, leadership is still concentrated within a small number of high-capacity academic and governmental research organisations, according to the calculated Herfindahl–Hirschman Index (HHI 0.086).

In addition to the bibliometric indicators reported above, a summary of the leading publication sources was prepared. Table 1 presents the Top 5 journals during the period 2021–2024, along with their total publications (TP), total citations (TC), 2024 Cite Score, the most cited article in 2024, the number of times it was cited, and the corresponding publisher. This table provides a concise overview of the journals that most significantly contributed to the dissemination of research in this field.

**Table 1:** Top 5 journals and most cited articles for 2024

Journal	TP (2021 2924)	TC	Cite score (2024)	The most cited article (2024)	Time cited	publisher
Frontiers in Immunology	24,712	268,044	10.8	Medicinal plants: bioactive compounds, biological activities, combating multidrug-resistant microorganisms, and human health benefits - a comprehensive review	41	Frontiers Media SA
Cancers	22,133	194,180	8.8	Glioblastoma: Clinical Presentation, Multidisciplinary Management, and Long-Term Outcomes	50	Multidisciplinary Digital Publishing Institute (MDPI)
International Journal of Molecular Sciences	59,508	537,397	9.0	Type 2 Diabetes Mellitus: New Pathogenetic Mechanisms, Treatment and the Most Important Complications	97	Multidisciplinary Digital Publishing Institute (MDPI)
Frontiers in Oncology	20,973	145,364	6.9	Explainable AI in medical imaging: an interpretable and collaborative federated learning model for brain tumor classification	22	Frontiers Media SA
Journal for ImmunoTherapy of Cancer	1,928	34,264	17.8	Novel post-translational modification learning signature reveals B4GALT2 as an immune exclusion regulator in lung adenocarcinoma	57	BMJ Publishing Group

### Analysis of keywords

**Fig. 11:**
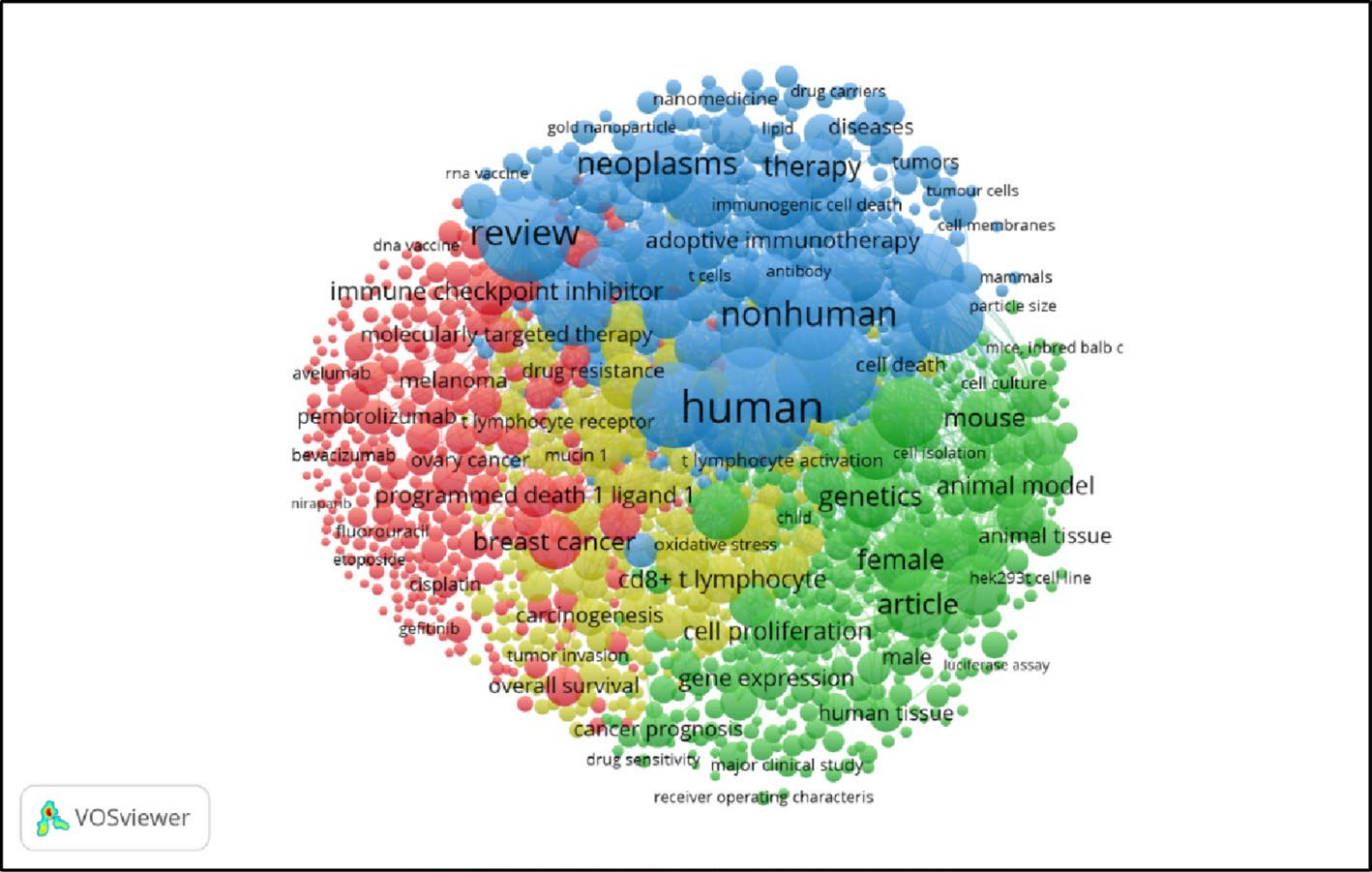
Co-occurrence network visualization of author keywords generated using VOSviewer (LinLog/modularity)

**Fig. 12:**
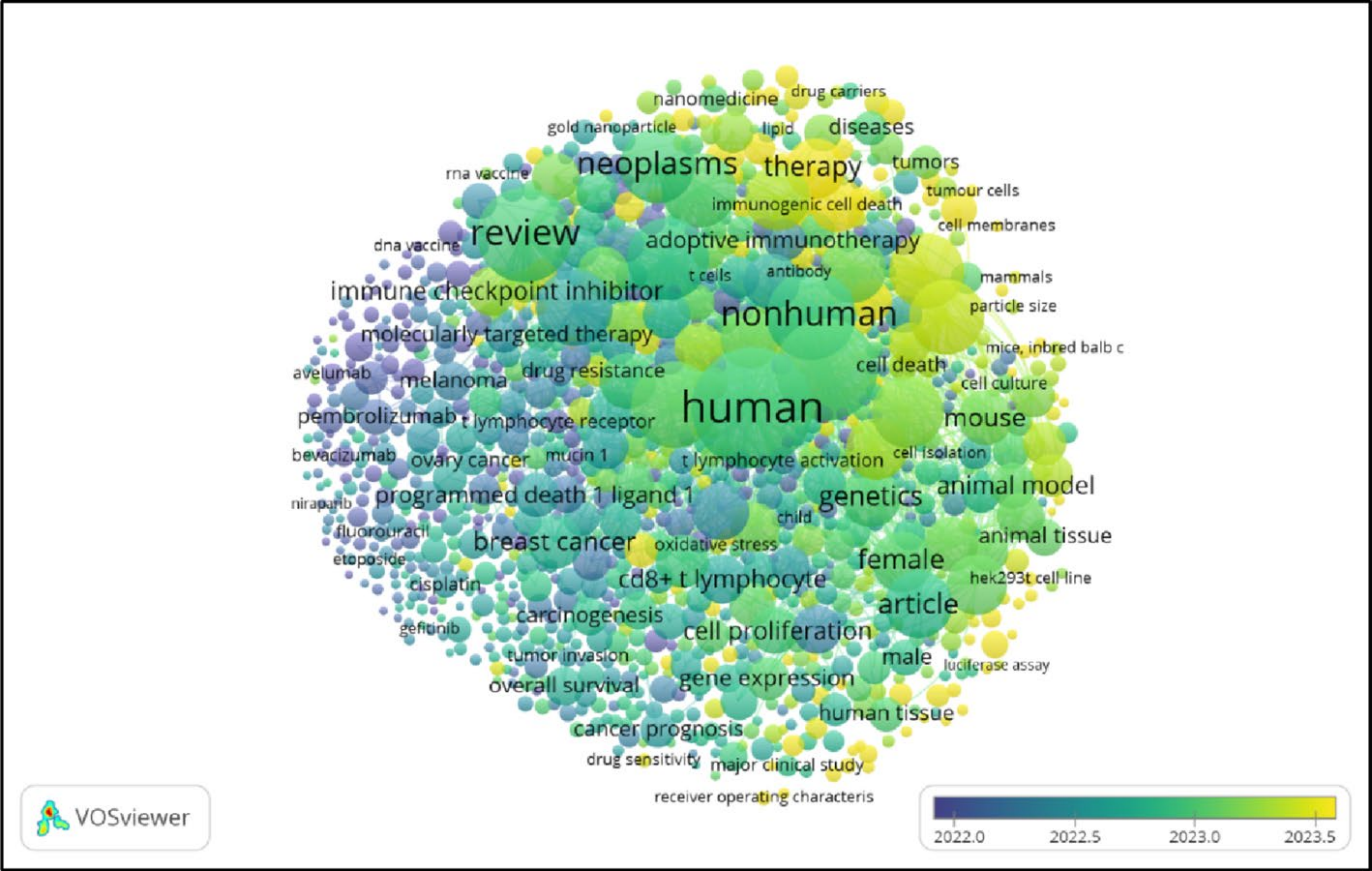
Co-occurrence overlay map of author keywords generated using VOSviewer (LinLog/modularity)

The parameters of VOSviewer were as follows: approach (Linlog/modularity) and a minimum of 10 keyword occurrences. Out of the 38,252 keywords, 3944 of them satisfied the requirements. The overall strength of co-occurrence linkages with other keywords was computed for each of the 3944 keywords. The keywords with the highest total link strength were chosen. The co-occurrence relationships among the most frequent keywords are illustrated in Fig. 11, while the temporal evolution of these keywords is shown in the overlay visualization in Fig. 12.

Four main subject clusters that show the development and multidisciplinary reach of cancer immunotherapy research over the study period are found by analysing the VOSviewer keyword co-occurrence network.

The main study theme, which focuses on human, nonhuman, neoplasms, and therapies, is represented by the blue cluster. With a focus on adoptive immunotherapy, immunogenic cell death, nanomedicine, and drug carriers, this cluster includes foundational and translational research that combines clinical trials with preclinical models. Strong bidirectional translation between experimental and clinical research is highlighted by the frequent use of “human” and “nonhuman.”

Key phrases like immune checkpoint inhibitor, programmed death-1 ligand 1, pembrolizumab, avelumab, and molecularly targeted therapy are included in the red cluster, which focuses on immune checkpoint inhibitors and targeted cancer treatments. The clinical growth of immunotherapy across many tumor types and the growing emphasis on treatment resistance and overall survival are indicated by disease-specific terms such as melanoma, breast cancer, and ovarian cancer.

The green cluster, containing terms such as mouse, animal model, genetics, gene expression, cell proliferation, and human tissue, also primarily represents molecular biology and experimental models. This cluster illustrates the value of in vitro and in vivo systems for elucidating immunological mechanisms, biomarker discovery, and therapeutic validation.

Some of the terms in the yellow cluster include CD8 + T lymphocyte, T lymphocyte activation, carcinogenesis, oxidative stress, and cancer prognosis, and they connect biological mechanisms with clinical implications. This group reports a growing interest in survival, prognostic markers, and the immunomodulation of tumors.

In conclusion, the keyword network shows a development from fundamental and animal-based research to immunotherapeutic approaches that are clinically applicable. Between 2011 and 2025, adoptive immunotherapy and immune checkpoint blockade have become major areas of scientific interest.

## Discussion

This bibliometric analysis provides a structured overview of the global scientific landscape of adoptive cell therapy (ACT) in triple-negative breast cancer (TNBC) over the period 2010–2025. These results indicate that research activity in this area has increased substantially, with a strong acceleration noted in the period following 2020. This temporal growth pattern suggests that ACT in TNBC has evolved from a niche and exploratory research area into a rapidly expanding translational domain. The increase in publication output likely represents a convergence of many factors, including the continued unmet therapeutic need in TNBC, the larger momentum around cancer immunotherapy, and advancements in cellular engineering and tumor immunobiology.

One of the most notable findings of the present analysis is the exponential increase in annual publications, reflecting both increasing academic interest and the conceptual maturation of the field. In bibliometric terms, rapid publication growth is often characteristic of an emerging discipline entering an accelerated phase of development. In the context of ACT for TNBC, this likely reflects a shift from foundational proof-of-concept work toward more translational and clinically oriented investigations. But growth in publication volume does not necessarily equal clinical maturity. Instead, the difference between publication growth and still-limited clinical implementation suggests scientific activity but translational constraint in the field. This discrepancy is significant because it suggests that the literature is outpacing achievements of durable therapeutic success in practice.

The geographical and institutional distribution of publications further clarifies how the field is evolving. Research productivity was highly concentrated, with China and the United States accounting for the majority of the total output, while a relatively small number of institutions and authors contributed disproportionately to the literature. From a bibliometric perspective, such concentration is often observed in technically demanding and resource-intensive research domains. ACT development requires advanced immunology platforms, cell manufacturing infrastructure, bioengineering expertise, and sustained financial investment; therefore, it is unsurprising that scientific leadership remains clustered within high-capacity research environments. This pattern suggests that progress in ACT for TNBC is currently being shaped by a limited but influential network of countries, institutions, and investigators.

At the same time, this concentration has important implications for the field’s intellectual structure. The presence of a small but highly productive author and institutional core suggests that knowledge production is being driven by specialized research hubs that may strongly influence topic prioritization, methodological approaches, and translational direction. While this can accelerate innovation by concentrating expertise, it may also limit diversity in research perspectives and contribute to thematic or geographic imbalance. Thus, the observed bibliometric concentration not only reflects scientific leadership but also highlights the need for broader international collaboration and more globally distributed translational capacity.

Keyword co-occurrence and clustering analyses offer additional insight into how the field’s conceptual focus has changed over time. The network structure indicates that early work was largely centered on foundational immunology, experimental models, and broad antitumor mechanisms, whereas more recent publications increasingly emphasize clinically relevant themes such as immune checkpoint inhibition, tumor microenvironment modulation, metabolic reprogramming, biomarker discovery, and combination immunotherapy strategies. This thematic transition is particularly important because it reflects an evolution from asking whether ACT can be theoretically applied to TNBC to address why its clinical effectiveness remains limited and how it may be improved.

In this regard, these bibliometric findings represent more than mere productivity mapping; they effectively reflect the scientific response to translational challenges. The increasing frequency of terms related to metabolism, immune suppression, stromal biology, and biomarkers is a strong indication that the field is gradually pivoting around the central biological barriers limiting ACT efficacy in solid tumors. Such a thematic evolution is highly congruent with the broader TNBC immunotherapy literature and indicates growing sophistication in how researchers model therapeutic resistance.

Indeed, one of the major translational challenges reflected in this literature is the biological heterogeneity of TNBC. High inter- and intratumoral heterogeneity complicates the identification of stable and uniformly expressed therapeutic targets, thereby limiting the durability of antigen-directed ACT approaches [15]. This problem is further intensified by lineage plasticity and epithelial–mesenchymal transition (EMT), which can dynamically alter tumor-cell identity and surface antigen expression over time [16]. EMT-associated transcriptional programs may reduce target antigen visibility while simultaneously promoting more stem-like and immune-evasive phenotypes, thereby contributing to immune escape and treatment resistance [17]. The increasing prominence of mechanistic and biomarker-related themes in recent keyword networks likely reflects the field’s growing recognition that durable ACT efficacy in TNBC depends not only on engineering better cells but also on understanding the tumor’s evolving biology.

An indirect keyword analysis also revealed an increasingly notable focus on the tumor microenvironment (TME). The TNBC microenvironment harbors numerous mechanisms of immune suppression, including regulatory T cells, myeloid-derived suppressor cells, inhibitory cytokines, and immune checkpoint pathways, all of which may affect the persistence and function of adoptively transferred therapeutic lymphocytes [18]. In addition to classical immune suppression, the TME in TNBC imposes substantial metabolic stress, including hypoxia, lactate accumulation, extracellular acidosis, and nutrient competition, all of which can diminish T-cell proliferation, cytokine production, and cytotoxic function [19–21]. The bibliometric rise of themes related to metabolism and microenvironmental regulation, therefore, appears to reflect a genuine conceptual shift in the field, from a cell-centered engineering paradigm toward a more integrated systems-level understanding of ACT performance in solid tumors.

The thematic evolution observed in this analysis also aligns with the distinct challenges associated with individual ACT platforms. TCR-engineered T-cell approaches, for example, are constrained by dependence on major histocompatibility complex (MHC)-mediated antigen presentation, which is frequently dysregulated or lost in TNBC and can reduce therapeutic efficacy [22]. In addition, although TNBC may exhibit a moderate mutational burden, many candidate neoantigens are subclonal, transiently expressed, or highly individualized, thereby limiting broad clinical applicability and complicating therapeutic design [23, 24]. Similarly, tumor-infiltrating lymphocyte (TIL)-based therapy may be limited in TNBC by both quantitative and qualitative factors, including low baseline infiltration and the presence of exhausted or non–tumor-specific lymphocyte populations [25–27]. These limitations help explain why keywords related to immune dysfunction, biomarkers, and T-cell fitness are becoming increasingly relevant in the literature.

Comparable patterns are seen in the evolution of CAR-T research, which appears to be shifting from simple antigen-targeting strategies toward more complex engineering approaches to improve trafficking, persistence, specificity, and safety. In TNBC, CAR-T development is hindered by poor infiltration into solid tumor tissue, stromal barriers, antigen heterogeneity, and the absence of truly tumor-exclusive surface targets [28, 29]. Antigens such as ROR1 and TnMUC1 are promising but not entirely tumor-restricted, raising the risk of on-target/off-tumor toxicity [25, 30]. The increasing bibliometric prominence of terms related to multi-targeting, biomarkers, combination therapy, and resistance mechanisms likely reflects attempts to address these limitations through more sophisticated therapeutic design.

Collectively, the bibliometric results imply that research in ACT within TNBC is no longer focused solely on feasibility. Instead, it now seems to be moving into a more mature translational phase of research with increasing scientific focus on optimization, patient selection, resistance biology, and multimodal integration. Thematic shifts of focus towards biomarker-driven strategies, chemokine receptor engineering, stromal modulation, and combinatory regimens with checkpoint blockade or other immune-modulating approaches are recent examples that support this argument [31]. These developments indicate that future progress is likely to depend less on any single ACT platform in isolation and more on how effectively these therapies can be adapted to TNBC’s biological and microenvironmental realities.

Overall, this study shows that bibliometric analysis can yield more than a simple descriptive count of publications: it can reveal how a field is intellectually articulated, where scientific leadership concentrates, and how research priorities shift in response to translational needs. For ACT for TNBC specifically, the literature demonstrates a field that is both rapidly expanding and simultaneously confronts the biological complexity of one of the most challenging solid tumors to treat. This gravitational shift towards tumor-deliverable, biology-driven engineering, biomarker discovery, and microenvironment-oriented strategies indicates an evolving research ecosystem that increasingly resonates with the demands for clinically relevant therapeutic advancement.

### Limitations

This bibliometric study has limitations that should be noted when interpreting the findings. First, the analysis was conducted solely on the Scopus database; therefore, publications indexed in other databases, such as Web of Science, PubMed, or Embase, might not have been captured. Second, the search was limited to English-language publications, which might introduce language bias and miss applicable studies published in other languages. Third, only articles and review articles were included; other document types, such as conference papers, editorials, letters, and book chapters, were excluded. This approach facilitates consistency but might exclude potentially relevant scholarly contributions.

Furthermore, the current analysis did not differentiate between original research and review articles in every bibliometric assessment, which may have introduced some analytical bias, particularly in citation-based and productivity-related interpretations. Citation counts might also inherently favor older publications, as the former have had more time to accrue citations than newer works. Also, bibliometric analysis relies on the metadata held in databases being accurate and complete (such as author names, affiliations, institutional information, or any indexed keywords), which can sometimes lead to inconsistencies or errors during indexing.

Despite these limitations, the present study provides a broad and informative overview of the scientific development, thematic evolution, and global research structure of ACT in TNBC. The findings should therefore be interpreted as a robust representation of the indexed literature rather than as an exhaustive account of all research activity in the field.

### Future directions

The bibliometric trends identified in this study suggest that future research on ACT for TNBC may need to address the biological and translational challenges highlighted by the current knowledge structure of this field. Notably, the temporal evolution of keywords demonstrates an increasing trend towards tumor microenvironment modulation, biomarker-driven patient stratification, metabolic reprogramming, and combination immunotherapeutic modalities.

Thus, future studies should focus on identifying more stable/tumor-restricted antigen targets, developing multi-target and resistance-adapted cellular constructs, and utilizing ACT in combination with complementary treatment strategies, including immune checkpoint blockade, stromal-targeting approaches, and metabolic interventions. More emphasis should also be placed on the discovery and validation of predictive biomarkers to refine patient selection and more accurately characterize which TNBC subpopulations are most likely to benefit from ACT-based approaches.

In terms of research structure, the strong concentration of publications among a small number of countries and institutions could indicate that broader international collaboration and more inclusive translational research networks will be needed to boost progress and improve reproducibility across healthcare and research settings. Finally, both specific filters by document type and database comparisons, as well as more sensitive assessments of thematic evolution, can aid in presenting our understanding of how this field is evolving in future bibliometric investigations.

Overall, progress in ACT for TNBC will greatly benefit from further convergence among cell engineering, tumor immunobiology, and precision oncology, supported by increasingly collaborative, clinically driven research efforts.

## Conclusion

This bibliometric study aims to provide a world-scale overview of research on ACT in TNBC over the last 10 years. The results demonstrated an exponential increase in publication output, particularly after 2022. This expansion mirrors the rapid and robust evolution of immunotherapeutic modalities for this aggressive and recalcitrant breast cancer subtype. Research in this field is extremely concentrated in several countries, institutions, and high-impact authors. This focus, which constantly returns to expertise, infrastructure, and resources for knowledge development, is central.

Despite growing publications, beyond biological and technical constraints common to any solid tumor, the clinical application of ACT in TNBC remains limited. Analysis of keywords and themes shows an apparent shift from basic science to clinical implications. Such strategies include CAR-T cells, TIL therapy, genetically modified (engineered) T cells, tumor microenvironment modulation, metabolic reprogramming, and immunotherapies guided by biomarkers. This change is driven by the increasing recognition that intra-tumoral heterogeneity, immunosuppressive historical microenvironments, a metabolically unfriendly environment, antigenic variation, and safety issues are all limiting factors for durable therapeutic outcomes in TNBC.

It is interesting to note that the long-term research trends also suggest that the field is no longer a single modality-dominant field. Currently, it is moving toward rational combinations and next-generation engineering to enhance efficacy, persistence, and safety. The increasing emphasis on biomarkers, patient selection, and tumor biology–driven antigen discovery suggests a more refined state of the art. There is also a greater understanding of what is required for successful ACT deployment in TNBC.

## Data Availability

The data analyzed in this bibliometric study were retrieved from the Scopus database (Elsevier). Scopus is publicly accessible at: https://www.scopus.com. No accession numbers are applicable, as the data consist of bibliographic records extracted from a commercial literature database. The datasets generated and analyzed during the current study are available from the corresponding author upon reasonable request.
